# Lymphatic Function of the Lower Limb after Groin Dissection for Vulvar Cancer and Reconstruction with Lymphatic SCIP Flap

**DOI:** 10.3390/cancers14041076

**Published:** 2022-02-21

**Authors:** Anna Amelia Caretto, Gianluigi Stefanizzi, Simona Maria Fragomeni, Alex Federico, Luca Tagliaferri, Valentina Lancellotta, Giovanni Scambia, Stefano Gentileschi

**Affiliations:** 1 Dipartimento di Medicina e Chirurgia Traslazionale, Università Cattolica del Sacro Cuore, 00168 Rome, Italy; carettoaa@virgilio.it (A.A.C.); stefano.gentileschi@unicatt.it (G.S.); 2 Dipartimento Scienze della Salute della Donna, del Bambino e di Sanità Pubblica, Fondazione Policlinico Universitario A. Gemelli IRCCS, 00168 Rome, Italy; gianluigi.stefanizzi@policlinicogemelli.it (G.S.); simona.fragomeni@policlinicogemelli.it (S.M.F.); alex.federico@policlinicogemelli.it (A.F.); 3 Dipartimento di Diagnostica per Immagini, Radioterapia Oncologica ed Ematologia—Fondazione Policlinico Universitario A. Gemelli IRCCS, 00168 Rome, Italy; luca.tagliaferri@policlinicogemelli.it (L.T.); valentina.lancellotta@policlinicogemelli.it (V.L.)

**Keywords:** vulvar cancer, gynecologic cancer, groin dissection, inguinal lymphadenectomy, inguinofemoral lymphadenectomy, lymphedema, lymphatic flap, SCIP flap, lymphedema prevention, personalized medicine

## Abstract

**Simple Summary:**

Inguinal lymphadenectomy for vulvar cancer is often followed by secondary lower limb lymphedema. This significant treatment-related morbidity cannot be healed and worsens over time, causing symptoms significantly affecting the patients’ quality of life and creating disability, economic burden for physical therapies, infections and reduction of presence and performance at work. This study aimed to reveal the incidence and severity of lower limb lymphedema in patients who have undergone inguinal lymphadenectomy and immediate groin reconstruction with the Lymphatic Superficial Circumflex Iliac Perforator flap. In our series of patients, a significant protective effect of the lymphatic SCIP flap-based immediate reconstruction of the inguinal area emerged, and our results suggest that this easy and quick technique can reduce the incidence and severity of secondary lower limb lymphedema after groin dissection for vulvar cancer.

**Abstract:**

Inguinofemoral lymphadenectomy, frequently performed for vulvar cancer, is burdened with substantial immediate and long-term morbidity. One of the most disabling treatment-related sequelae is lower limb lymphedema (LLL). The present study aims to describe the wound complications and the severity of LLL in patients who have undergone groin dissection for vulvar cancer and immediate inguinal reconstruction with the Lymphatic Superficial Circumflex Iliac Perforator flap (L-SCIP). We retrospectively reviewed the data of patients who underwent bilateral groin dissection and unilateral inguinal reconstruction with the L-SCIP. The presence and severity of postoperative LLL during the follow-up period were assessed by lymphoscintigraphy and limbs’ volume measurement. In addition, immediate complications at the level of the inguinal area were registered. The changes between preoperative and postoperative limb volumes were analyzed by Student’s *t* test. *p* values < 0.05 were considered significant. Thirty-one patients were included. The mean variation of volume was 479 ± 330 cc3 in the side where groin reconstruction had been performed, and 683 ± 425 cc3 in the contralateral side, showing smaller variation in the treated side (*p* = 0.022). Lymphoscintigraphy confirmed the clinical findings. Based on our results, inguinal reconstruction with L-SCIP performed at the same time of groin dissection in patients treated for vulvar cancer can provide a significant protective effect on LLL.

## 1. Introduction

The mainstay treatment of vulvar cancer is wide local excision with uni- or bilateral groin dissection. Despite many studies demonstrating feasibility and excellent results of sentinel node biopsy in early stages, in more than 50% of the cases, inguinofemoral lymphadenectomy is still performed.

Beyond the superficial lymph nodes, a lymphadenectomy should remove the deep inguinal lymph nodes, because leaving the lymphatic tissue medial to the femoral vein can worsen patient prognosis [1,2]. This aggressive resection of lymphatic and adipose tissue, associated with groin skin flaps undermining, wide dead space and femoral vessels exposure, creates an impressive treatment-related morbidity, making the inguinofemoral lymphadenectomy one of the surgical procedures with the highest risk of immediate and long-term complications [3].

Wound breakdown, infection and lymphatic leakage are the most frequent short–term complications, while lymphedema and erysipelas/lymphangitis are the most frequent late complications. Sartorius transposition, videoendoscopic assisted lymphadenectomy, modified surgical incisions, fascia preservation and robotic surgery have been proposed to reduce the risk of postoperative complications, but none of these showed a significant reduction in morbidity [4,5,6,7,8,9].

Reconstructive procedures and microsurgery seem to be able to reduce lower limb lymphedema (LLL) risk, but several authors conclude that further studies are needed to confirm these results [10,11,12,13].

In 2017, we described a modification of the Superficial Circumflex Iliac Perforator flap, including the lymphatic vessels of the flank (L-SCIP), for the reconstruction of the inguinal region after groin dissection for vulvar cancer [14]. This flap can cover both cutaneous and subcutaneous inguinal defects [15], filling the dead space that follows from the lymphadenectomy with tissue rich in lymphatic vessels. In a small series of patients, we observed a reduced incidence of secondary lymphedema in the limbs reconstructed with this flap, and postoperative lymphoscintigraphy showed a rerouting of the lymph flow through the flap. 

The present study aims to describe the short-term complications and the long-term incidence and severity of LLL in patients who have undergone groin dissection for vulvar cancer and immediate reconstruction of the inguinal area with the L-SCIP.

## 2. Materials and Methods

We performed a retrospective observational study, reviewing the data collected in the clinical records and during the follow-up of all patients who underwent bilateral groin dissection for vulvar cancer and reconstruction of one of the two the inguinal areas with the L-SCIP in our institution between 2015 and 2021. The study protocol was approved by the Institutional Review Board (IEC of the Fondazione Policlinico Universitario Agostino Gemelli IRCCS; ID Approval: 3335) and adhered to the STROBE guidelines. 

Before surgery, the need of reconstruction of the inguinal area was considered when one of the following conditions was probable: relevant subcutaneous dead space after aggressive resections in patients with thick fat layer [16,17,18]; the need to resect inguinal skin creating a coverage defect or the possibility of thin inguinal skin flaps after undermining and resection of the underlying adipose and lymphatic tissue; superficial lymphadenopathies with relevant risk of marginal necrosis and femoral vessels exposure in case of wound breakdown. After completion of lymphadenectomy, the possible indication was re-evaluated and, where appropriated, confirmed. The patients requiring bilateral inguinal reconstruction, according to these indications, were excluded from the study. Patients with pre-operative signs of lower limb swelling or lymphedema or those who had undergone previous inguinal surgery were excluded as well.

The variables of interest that we collected were the patients’ age, height, weight, BMI, smoking habits, comorbidities, previous surgery, cancer site and histological features, anatomical subunits involved by the resection, flap size, features of the vascular pedicle, any other simultaneous reconstructive procedure, wound complications, and months of follow-up. To reduce the possibility of errors in the data detection and registration, we performed a double check, whenever possible, by comparing the data present in the digital clinical archive of our institution, which are available for outpatient and hospitalized patients, and in Redcap, a platform available in our hospital for online databases and surveys. 

The primary outcome measure of this investigation was the change of limb volume after surgery during the follow-up period. Secondary outcome measures were the occurrence of immediate complications at the level of the inguinal area in terms of wound dehiscence requiring revisional surgery, lymphatic leakage, according to the classification of Gerken et al. [19], and infections of the inguinal surgical sites during the first 3 months of the follow-up period.

To preoperatively identify the patients at high-risk for postoperative lymphedema, for which we applied a specific strict surveillance protocol, we routinely evaluated, before surgery, the lymphatic function in every patient undergoing groin dissection for vulvar cancer by studying both drainage function and by measuring the limbs. Drainage function was analyzed by lymphoscintigraphy performed according to a method widely reported in the literature, which we routinely employ for lymphedema and lymphocele study [20,21,22]. Lymphoscintigraphy was repeated not before 12 months after surgery in those patients that showed signs of lymphatic swelling in one of the two limbs during the follow-up, according to the guidelines of the International Society of Lymphology [23]. The data recorded were the course of lymph flow, the Transport Index [24] and possible extravasation and dermal backflow areas. Measurements of the limbs were determined getting the girths every 4 cm, from the tip of the foot to the origin of the thigh. Both mean circumference and volumes were calculated. Limb volumetry was obtained by the frustum formula mentioned by Casley-Smith [25] which is as follows: V = (h)(C2 + Cc + c2)/12(π), where “C” is the girth measurement of distal section, “c” is the girth measurement of the proximal section and “h” is the distance between distal and proximal section. Limb measurements, with calculation of volume and mean circumference, were repeated every 6 months after surgery during the follow up visits. Pre- and postoperative photographic documentations were taken. After surgery, no bandage or compressive garment was prescribed to the patients. Patients did not undergo any conservative measure to prevent lymphedema during follow up. The side where the lymphatic flap was employed to reconstruct the inguinal area was considered as the “treated side”, while the contralateral was considered as the “untreated side”.

The wound complications considered were infection, dehiscence, and skin edge necrosis. In the immediate postoperative period, the amount of groin drainage was registered. The lymphatic leakage was classified into four grades, grade A: a drainage output of lymphatic fluid from the drains of at least 50 mL/24 h for more than 5 but less than 10 postoperative days or the appearance of a seroma without the occurrence of wound complications or need for secondary surgery; grade B: prolonged leakage of lymphatic fluid from the drains of at least 50 mL/24 h for at least 10 postoperative days or by the appearance of seromas after an initial removal of the drains that required interventions; grade C: lymphatic leakage leading to reoperation or causing any delay of further treatment; the remaining patients had no lymphatic leakage and were classified as grade 0. The surgical technique employed for flap harvesting and for reconstruction has already been described in our previous publications [14,15,26]. 

### Statistical Analysis

The sample was described in its clinical and demographic features using descriptive statistics techniques. Quantitative variables were described using the following measures: minimum, maximum, range, mean and standard deviation. Qualitative variables were summarized with absolute and percentage frequency tables. Normality of continuous variables was checked using Kolmogorov–Smirnov test. All values of pre- and postoperative MC and volume were reported as the mean ± SD. The changes between preoperative and postoperative values of limb measurements were analyzed by Student’s *t* test. *p* values < 0.05 were considered significant. All statistical analyses were performed with IBM^®^SPSS^®^Statistics software V.24.

## 3. Results

Between 2015 and 2021, 31 patients underwent vulvar cancer resection, bilateral groin dissection, and reconstruction of one of the two inguinal regions with a pedicled L-SCIP. 

The patients’ ages ranged from 38 to 87 years, with a mean of 70 ± 10 years. Mean BMI was 27. Six patients were smokers. Seven patients were obese, and 10 were overweight. 

Twenty-nine patients had squamous cell carcinoma, two had invasive Paget’s disease. In all patients, ablative surgery of vulvar cancer required bilateral groin dissection worthy of reconstruction in one of the two sides; specifically, 10 patients showed only a subcutaneous wide dead space with femoral vessel exposure, and 21 required skin coverage of the groin area. In twelve patients, the reconstruction was performed in the right groin, while in nineteen in the left. The flap size ranged from 32 (8 × 4 cm) to 330 cm^2^ (30 × 11 cm) with a mean flap size of 127 cm^2^. In fourteen flaps, only the deep branch of the SCIA was employed as a pedicle, while in 10 flaps, only the superficial branch was employed. In seven flaps, both the superficial and deep branches were included. In one case, two separated defects were present in the inguinal and mons pubis areas, and we harvested a chimeric flap employing both the superficial and deep branches to reconstruct the two defects independently. The number of lymph nodes removed from the groin ranged from 6 to 15 (mean 9.2). The mean extra operative time for flap harvesting and inset was 75 min. The mean follow up was 30 months. 

For each patient, the differences between the preoperative and postoperative volumes of the treated and untreated limb were calculated. The mean variation of volume was 479 ± 330 cc3 in the treated side, and 683 ± 425 cc3 in the untreated side. This difference was statistically significant (*p* = 0.022), showing a smaller variation in the treated side. 

**Figure 1:**
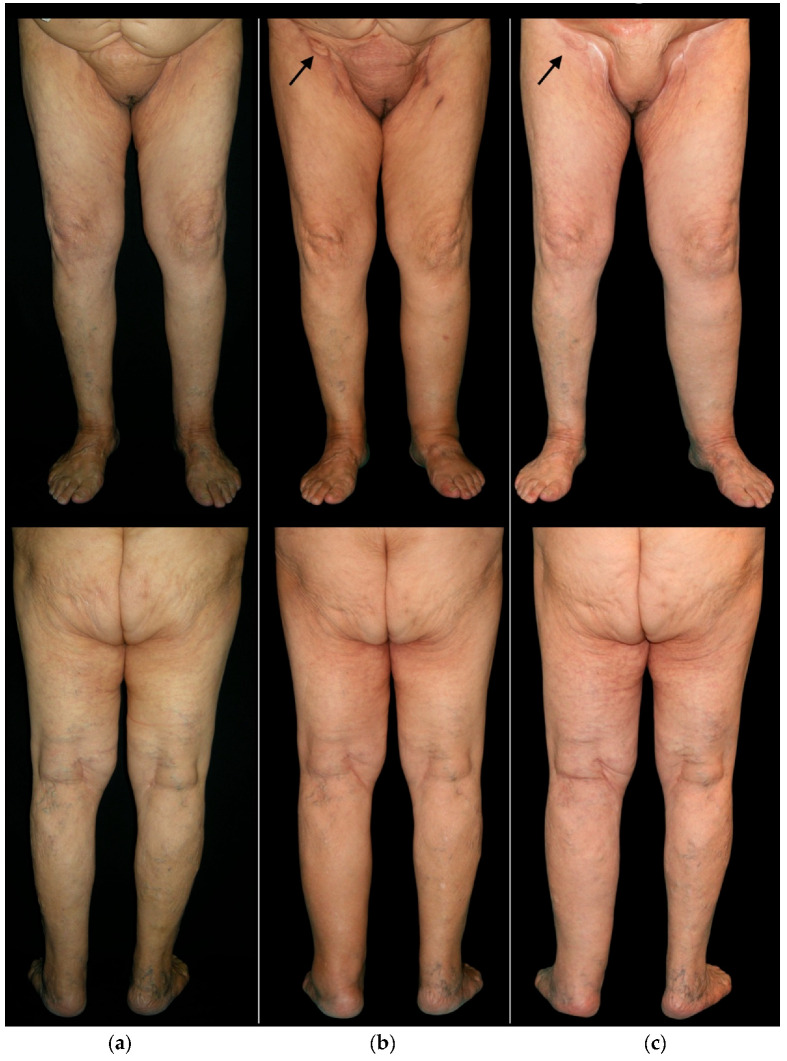
(**a**) Frontal and posterior pre-operative view of an 80-year-old woman undergoing surgery for vulvar cancer, involving bilateral groin dissection and reconstruction of the right inguinal area with L-SCIP. (**b**) Frontal and posterior view of the same patient one year after surgery. The black arrow indicates the flap skin island at the level of the right groin. The patient shows moderate swelling of the left lower limb (untreated side) and mild swelling of the right limb. (**c**) The same patient, 4 years after surgery, shows severe swelling of the left lower limb and no swelling of the right limb.

**Figure 2:**
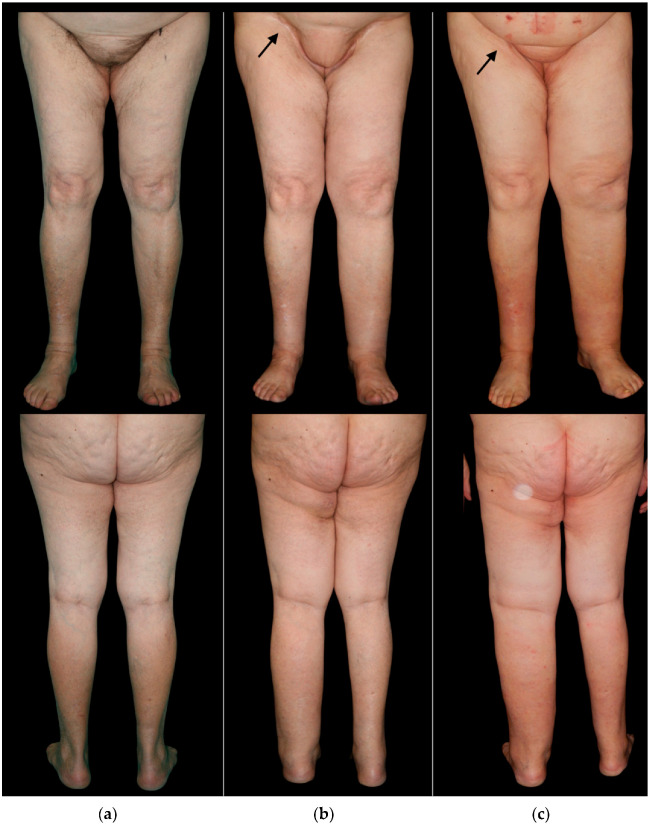
(**a**) Frontal and posterior pre-operative view of a 77-year-old woman undergoing surgery for vulvar cancer, involving bilateral groin dissection and reconstruction of the right inguinal area with L-SCIP. (**b**) Frontal and posterior view of the same patient one year after surgery. The black arrow indicates the flap skin island at the level of the right groin. The patient shows moderate swelling of the left lower limb (untreated side) and mild swelling of the right lower limb. (**c**) The same patient, 5 years after surgery, shows severe swelling of the left lower limb and mild swelling of the right lower limb.

Six patients showed no clinical signs of swelling with no difference between the treated and the untreated side. Twenty-five patients showed pitting edema at the level of the untreated side (Figure 1), and eight in the treated side (Figure 2). 

Only five treated limbs showed an increase in volume >10%, while 14 untreated limbs showed an increase > 10%. The mean percentage of increase in the limb volume was 10.4% ± 7% in the untreated side and 7.2% ± 5% in the treated side, with a significant difference provided by the presence of the flap (*p* = 0.013). Two patients showed a larger volume increase in the treated side. In both these cases, the patients had also undergone a pelvic lymphadenectomy, together with groin dissection, in the side of the flap. 

All patients showing clinical signs of swelling were assessed by lymphoscintigraphy that confirmed the lymphatic drainage impairment, showing TI > 10 and lymph extravasation. In the untreated limb, all these patients showed diffuse dermal backflow, while two cases showed rerouting of the lymph flow through the groin toward the pelvis, and in two cases, complete stop of progression at the level of the leg. In the treated limb, we observed a combination of linear and ectasic lymphatic pathway in the leg with extravasation or ectasic pathway in the thigh. Some passage of the lymph to the pelvis, with visualization of the iliac nodes, was observed in all the treated limbs but four. 

**Figure 3:**
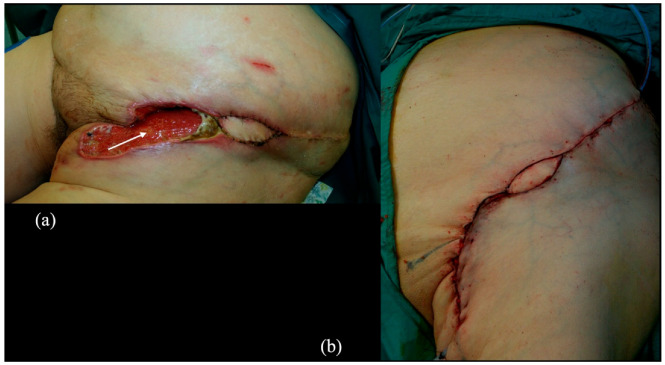
(**a**) Wound breakdown after groin dissection and reconstruction with L-SCIP flap at the level of the left inguinal area. The white arrow indicates the buried part of the flap, showing granulation tissue after VAC-therapy. The presence of the flap prevented femoral vessels exposure. (**b**) Postoperative view of the same patient after debridement and primary closure of the wound dehiscence.

Complications of wound healing occurred in 10 groins in the untreated side and in four groins in the treated side. We observed two relevant wound breakdowns at the level of the edges of lymphadenectomy cutaneous flaps in two obese patients; one occurred at the site of left groin dissection, with the presence of a buried lymphatic SCIP flap that avoided the exposure of femoral vessels (Figure 3) and healed by VAC therapy, debridement and primary closure, and the other in an untreated groin that required revision and reconstruction with pedicled ALT flap. 

**Figure 4:**
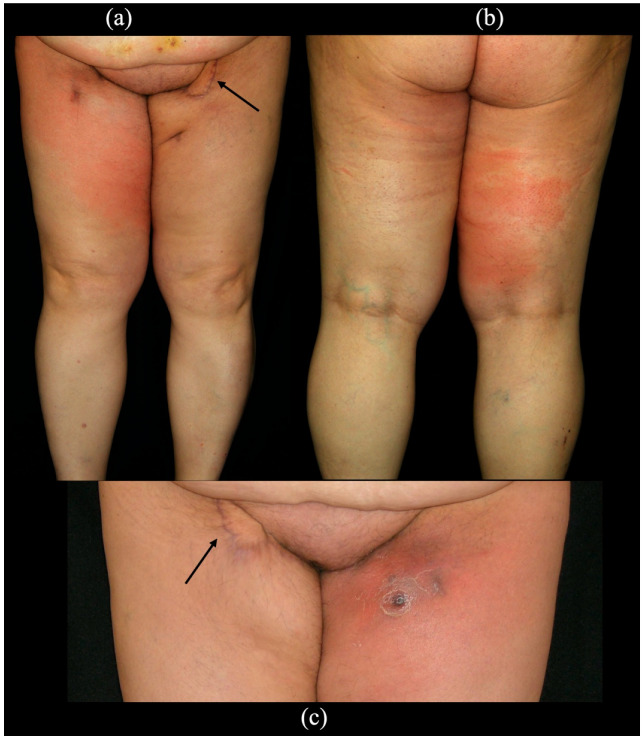
This picture shows the two patients of our series affected by postoperative infection. (**a**,**b**) Anterior and posterior view of a 57-year-old woman showing lymphangitis of the right lower limb (untreated side) involving the anterior and posterior surface of the thigh 3 months after bilateral groin dissection for vulvar cancer and reconstruction of the left inguinal area with L-SCIP. The black arrow indicates the skin island of the flap. This patient was healed by antibiotic therapy. (**c**) Postoperative fluid collection and infection at the level of the left inguinal area 1 month after bilateral groin dissection and reconstruction of the right inguinal area with L-SCIP. The black arrow indicates the skin island of the flap in the right groin. This patient was healed by antibiotic therapy and repeated fluid aspiration.

We observed two cases of infection, both in inguinal areas not treated by SCIP flap (Figure 4), healed by antibiotics and in one of the two cases also by repeated fluid aspiration. 

Wound healing complications did not cause a delay in adjuvant therapies in any of the cases. Drains were removed when the output was less than 30 mL/24 for 2 days. Five patients developed a prolonged secretion of lymphatic fluid, showing a drainage output of more than 30 mL/24 h, for more than 5 but less than 10 days in the treated side. Sixteen patients showed output of more than 30 mL/24 h for more than 10 days in the untreated side and one patient in the treated side. One patient developed a lymphatic cyst after drain removal in the untreated side and required a new drain placement under ultrasound guide.

## 4. Discussion

Inguinal lymphadenectomy is frequently undertaken for vulvar cancer and is burdened with a high risk of immediate and delayed complications. Morbidity after groin dissection is high regardless of the type of primary tumor. For example, comparable complication rates have been described for vulvar cancer and lower limb melanoma [27,28]: wound breakdown, 3–26%; wound infection, 9–30%; lymphocele, 5–46%; and lymphedema, 20–64% [29,30,31]. Different risk factors for the occurrence of wound complications have been described in previous publications, such as smoking, diabetes and overweight [32]. The wide dead space that occurs after surgery is the main cause of postoperative complications. Frequently, the collection of fluid in the surgical site is the first trigger of immediate complications such as seroma, lymphocele, infection, and wound breakdown. 

Possible preventive and therapeutic interventions over fluid collection such as fibrin glue, talcum, and Picibanil (OK- 432) are performed to cause obliteration of leaking lymphatic vessels [33,34,35]. Lymphatic vessel coagulation and the presence of relevant scarring of the dead space that remains after lymphatic and adipose tissue removal, which prevents the lymph flow from re-routing, are responsible for the high incidence of secondary lymphedema, namely, up to 70% [36], with the highest risk in cases of wound infection, older age, obesity, or adjuvant radiation therapy [37,38,39,40,41].

Preservation of the deep fascia [42], of the long saphenous vein [43], sartorius muscle transposition [6], modified course of surgical incision or video-endoscopic minimally invasive inguinal lymphadenectomy (VEIL) [44] are some of the modifications that have been proposed to the classical inguinal nodes dissection, initially described by Taussig [45] for vulvar carcinoma and modified by Daseler et al. in 1948 [46] and by Way et al. in 1960 [47]. Even though these techniques were developed to reduce complications without impairing oncological radicality, they showed inconclusive results in subsequent studies. A randomized controlled trial in patients with vulvar cancer showed that sartorius transposition did not reduce complications and perhaps increased seroma formation [6]. It has even been argued by some authors that sartorius transposition may increase the risk of persistent lymphedema [48]. Immediate flap-based reconstruction can reduce the complication rate carrying well-vascularized tissue to fill the dead space and decrease the possibility of seroma and subsequent infection [49]. Similar conclusions were drawn by other authors who advocated for the routine use of myocutaneous flaps following ilioinguinal dissection [50]. Flaps previously described to reconstruct groin defects include anterolateral thigh flap, abdominal flaps, gracilis, sartorius, and rectus femoris, with each showing its drawbacks and advantages [51,52]. Reconstruction with well-vascularized tissue, decreasing the risk of seroma and infection of the surgical site, can reduce the risk of secondary lymphedema because it has been demonstrated that these complications can be interrelated. Seroma has been assumed to originate from inflammatory lymphatic leakage [53], impairing wound healing and providing an entry for infection. Nevertheless, this reduction in LLL risk is not specifically caused by an improvement of lymphatic re-routing through the surgical site of lymphadenectomy accomplished by the flap, but to the general reduction of risk factors such as infection and lymphocele. Despite the fact that many flaps employed for inguinal reconstruction in vulvar cancer surgery can provide filling of the dead space [54,55], none of these has ever shown a specific role in improving lower limb lymphatic drainage function [50,52], except for those that can carry, in site, a huge amount of lymphatic tissue at the same time, providing a biological scaffold for lymphatic re-routing through the flap itself [12,14]. In addition, a theoretical effect of a lymphatic flap-based reconstruction over lymphatic leakage in a lymphadenectomy site has been supposed, arguing that after transfer, lymph can be absorbed into the flap directly from the distal stumps of the lymph vessels, reducing since the early post-operative period the wound discharge [56]. Nevertheless, in our series, the immediate complications in terms of wound dehiscence, infection and lymphatic leakage are too rare to draw conclusions. 

Conversely, the analysis of pre- and post-operative limb volume revealed a significant effect provided by the L-SCIP flap-based reconstruction over the risk of late secondary lymphedema. After groin dissection, every patient has a different susceptibility to develop or not LLL. Just to minimize the effect of the inter-subject variability on the study of the preventive effect of the flap, we included only patients undergone unilateral reconstruction and compared the reconstructed with the not reconstructed limb in the same patient.

Dealing with lower limb lymphedema diagnosis, the clinical system is the most common method used in the literature. In addition, we employed volume changes and lymphoscintigraphy to confirm the clinical diagnosis. Diagnosis based on self-reported symptoms of swelling, heaviness, or pain by the patient is not affordable because these symptoms are common after vulvar surgery, regardless of lymphedema status, and can lead to an overestimation of lymphedema incidence. 

In our study, some patients had a follow-up of more than 5 years, and the mean follow-up period was 30 months. We can consider it as a sufficient time to observe the preventive effect over lymphedema onset because we know that lymphedema most often occurs in the first 2 years following cancer treatment [57]. Moreover, in a recent meta-analysis about the surgical preventive approaches to lymphedema [58], of the 12 studies included, only 3 implemented a follow-up longer than 24 months. 

Lymphedema creates a dramatic burden for both patients and the healthcare system, and possible therapies can only improve symptoms such as heaviness, swelling, fluid stasis and infections, slowing down the progression of pathologic changes to both soft tissues and lymphatic vessels, but they cannot heal the disease that remains chronic and progressive [59]. Moreover, some specific anatomical sites of disease, such as genital lymphedema, are extremely intrusive in private life, and there is no consensus about the kind and timing of treatment [60]. Often, patients are confused and uncertain about possible surgical therapies because available information about benefits and side effects is lacking or inadequate [61]. For this reason, to reduce the risk of secondary lymphedema, many surgical protocols that associate lymphadenectomies with lymphatic flow restoration have been developed, based on lymphatic–venous anastomosis [58,59], lymphatic–venular anastomosis [62], or lymphatic flaps [26].

The advantages of L-SCIP are its low donor site morbidity, ease of harvesting and quick access to the vascular pedicle that can almost always be spared during groin dissection [14,26]. This retrospective observational study was undertaken to confirm our previous preliminary experience based on a small series of patients [14] that revealed a decrease in secondary lymphedema risk after groin dissection in patients who underwent simultaneous inguinal reconstruction employing L-SCIP flap. 

Although this study is not large enough to change the standard approach to groin dissection in patients at high risk of lymphedema, the number of patients for this specific population is substantial, and our findings are a relevant rationale for a randomized controlled prospective study, to confirm these results and provide stronger evidence. If these results will be confirmed, this technique could be considered as a quick and easy approach for patients undergoing groin dissection to reduce the incidence and severity of secondary lymphedema. 

The strengths of this study are the relevant number of cases included, with long-term follow-up, considering the rarity of patients operated on for vulvar cancer requiring bilateral groin dissection and unilateral flap-based inguinal reconstruction; and the case-control analysis performed comparing variations of limb volume in the same patient, decreasing the bias due to the intersubject variability to develop lymphedema or not and implementing the same postoperative management and follow-up schedule for “cases” and for “controls”. All flaps were executed by the same plastic surgeon (SG) expert in the field of vulvar and groin reconstruction. The main limitations are the retrospective and monocentric design.

## 5. Conclusions

This study supports the idea that the lymphatic SCIP flap is a safe, quick, and effective technique in achieving reconstruction of the inguinal area after groin dissection for vulvar cancer, with a relevant positive effect over incidence and severity of secondary lower limb lymphedema.

## Data Availability

The data presented in this study are available upon request from the corresponding author.
